# Comparative In Vitro Cytotoxicity of *Piper betle* Leaf Extracts in *KRAS* G12D-Mutant Pancreatic Cancer Cell Lines

**DOI:** 10.3390/cimb48090876

**Published:** 2026-08-29

**Authors:** Nur Syahirah Haidah Zahid, Aziemah Azizi, Aklimah Mustapa, Syahirah Shahlehi, Masayoshi Arai, Siti Nur Idayu Matusin

**Affiliations:** 1 Faculty of Halal Science and Sustainable Tourism, Universiti Islam Sultan Sharif Ali, Sinaut Campus, KM33, Jalan Tutong, Kampung Sinaut, Tutong TB1741, Brunei; 24mr2602@unissa.bn (N.S.H.Z.); jimah.azizi@gmail.com (A.A.); aklimah.mustapa@unissa.edu.bn (A.M.); 2 The University of Osaka ASEAN Campus Office Brunei Darussalam, No. 13, Kg Mabohai, Bandar Seri Begawan BA1111, Brunei; syahirah.shahlehi@unissa.edu.bn (S.S.); araim@phs.osaka-u.ac.jp (M.A.); 3 Faculty of Agriculture, Universiti Islam Sultan Sharif Ali, Sinaut Campus, KM33, Jalan Tutong, Kampung Sinaut, Tutong TB1741, Brunei; 4 Graduate School of Pharmaceutical Sciences, The University of Osaka, 1-6 Yamadaoka, Suita 565-0871, Osaka, Japan

**Keywords:** pancreatic cancer, *KRAS* G12D, *Piper betle*, cell viability, GC-MS, IC_50_, phenolic compounds, WST-8 assay

## Abstract

Pancreatic ductal adenocarcinoma (PDAC) is among the most lethal cancers worldwide, with limited treatment options and a high prevalence of oncogenic *KRAS* mutations. *Piper betle* contains numerous bioactive phytochemicals with reported anticancer activities, although its activity against pancreatic cancer remains incompletely characterized. This study evaluated the phytochemical composition of *P. betle* leaf extracts and the cell viability effects of ethanolic extracts against human *KRAS* G12D-mutant pancreatic cancer cell lines (PANC-1, AsPC-1, Panc 02.03, Panc 04.03, Panc 08.13 and Panc 10.05). Phytochemical screening, total phenolic content (TPC), total flavonoid content (TFC) and gas chromatography–mass spectrometry (GC-MS) analyses were performed to characterize the extracts. The ethanolic extracts exhibited higher extraction yield (17.26%), TPC (395.94 ± 0.010 mg GAE/g) and TFC (121.28 ± 0.009 mg QE/g) than aqueous extracts. GC-MS analysis tentatively identified phenylpropanoid compounds, including chavicol, chavibetol, and eugenol. Based on these findings, the ethanolic extract was selected for biological evaluation. Treatment significantly reduced cell viability in a concentration-dependent manner across all pancreatic cancer cell lines, with estimated IC_50_ values ranging from 21.54 to 54.91 µg/mL. These findings suggest that ethanolic *P. betle* leaf extract possesses promising biological activity and support further studies to identify its active constituents and elucidate their mechanisms of action.

## 1. Introduction

Pancreatic cancer, which develops from pancreatic exocrine or endocrine cells, remains a major global health burden, ranking sixth in cumulative mortality [1]. In 2022, approximately 510,992 new cases and 467,409 deaths were reported globally, reflecting the disease’s high mortality-to-incidence ratio [1,2,3]. Despite advances in cancer diagnosis and treatment, the five-year survival rate for pancreatic cancer remains below 13%, making it one of the most challenging cancers to treat [4]. Frequent occurrences are identified as pancreatic ductal adenocarcinoma (PDAC), which constitutes nearly 90% of cases [5].

The poor clinical outcome of PDAC is largely attributed to late-stage diagnosis, rapid disease progression, therapeutic resistance, and extensive tumor heterogeneity. Approximately 55% of PDAC patients are diagnosed at advanced or metastatic stages due to the lack of specific symptoms in early disease development [6]. Current therapeutic approaches, including chemotherapy regimens, such as gemcitabine and FOLFIRINOX, targeted therapy, and immunotherapy, have demonstrated limited effectiveness in improving long-term survival [7,8]. These challenges highlight the urgent need for alternative or adjunctive therapeutic strategies.

Oncogenic *KRAS* (Kirsten rat sarcoma 2 viral oncogene homolog) mutations are highly prevalent in PDAC and play a crucial role in tumor progression [9,10]. Among these, the *KRAS* G12D mutation is one of the most frequently reported and aggressive variants, representing approximately 45% of cases [11]. The mutation results from the substitution of glycine with aspartic acid at codon 12, leading to constitutive activation of *KRAS* signaling independent of growth signals [9]. Persistent stimulation of downstream pathways such as MAPK and PI3K/AKT promotes uncontrolled cell proliferation, survival, progression and metabolic reprogramming [12,13]. Although recent advances have led to the development of KRAS-targeted therapies, effective treatment options for *KRAS* G12D-mutant pancreatic cancer remain limited, prompting the exploration of novel therapeutic candidates.

Natural products, including plant-derived compounds, have gained increasing attention in cancer research due to their multi-targeted mechanisms and favorable safety profile [14,15,16,17]. Among these, *Piper betle*, commonly known as *Sirih hijau* in Southeast Asia, has been widely used in traditional medicine for its diverse pharmacological properties, including antimicrobial, anti-inflammatory, and anticancer activities [14,18]. Previous studies have demonstrated the presence of multiple phytochemicals such as phenylpropanoids, flavonoids and phenolic compounds in *P. betle*, which may contribute to its anticancer activity [19,20].

Although the anticancer potential of *P. betle* has been reported in several cancer models, studies evaluating its activity against pancreatic cancer, specifically against *KRAS* G12D-mutant pancreatic cancer, remain limited. Furthermore, information regarding the phytochemical composition and cell viability effect of *P. betle* extracts against *KRAS* G12D-mutant pancreatic cancer cell lines is scarce. Therefore, this study aimed to evaluate the in vitro activity of *P. betle* against *KRAS* G12D-mutant pancreatic cancer cell lines. A comprehensive panel of tests including phytochemical screening, total phenolic (TPC) and flavonoid (TFC) content, and GC-MS analysis were performed prior to an in vitro cell viability assay. The findings of this study provide preliminary insights into the potential of *P. betle* as a source of bioactive compounds for pancreatic cancer research.

## 2. Materials and Methods

### 2.1. Materials

Absolute ethanol, methanol, Folin–Ciocalteu reagent, sodium carbonate, aluminum chloride, gallic acid, quercetin, dimethyl sulfoxide (DMSO), and other analytical-grade chemicals and reagents used in this study were purchased from Sigma-Aldrich (St. Louis, MO, USA). Human *KRAS* G12D-mutant pancreatic cancer cell lines AsPC-1 (CRL-1682), Panc 02.03 (CRL-2553), Panc 04.03 (CRL-2555), Panc 08.13 (CRL-2551) and Panc 10.05 (CRL-2547) were acquired from the American Type Culture Collection (ATCC, Manassas, VA, USA). PANC-1 was obtained from RIKEN BioResource Research Centre (Tsukuba, Japan). Cell-culture reagents, Roswell Park Memorial Institute (RPMI)-1640 medium (with L-glutamine) and WST-8 colorimetric reagent were purchased from Nacalai Tesque, Inc. (Kyoto, Japan). Fetal bovine serum (FBS) was procured from Equitech-Bio Inc. (Kerrville, TX, USA). Antimycin A was obtained from LKT Laboratories, Inc. (St. Paul, MN, USA). Other chemicals used for the in vitro experiments were purchased from Sigma-Aldrich (St. Louis, MO, USA) or Kishida Chemical Co., Ltd. (Osaka, Japan).

### 2.2. Piper betle Leaf Collection

*P. betle* or *Sirih hijau* were originally obtained from a commercial plant supplier and subsequently cultivated under controlled conditions at the Herbs House Faculty of Agriculture, Universiti Islam Sultan Sharif Ali (UNISSA), Brunei. The plant species was identified as *P. betle* based on morphological characteristics. As the plant material was derived from commercially sourced cultivated stock, no wild collection was performed, and no voucher specimen was deposited. Leaves from the plant were harvested, cleaned with distilled water, and dried in a drying cabinet (Genlab, Widnes, UK) at 60 °C for three days. Once dried, the leaves were ground into a fine powder using a laboratory blender (Waring 800s, Stamford, CT, USA), and filter-sieved using a mechanical sieve (0.3 mm mesh hole size). The powder obtained was then weighed and stored in airtight containers at room temperature, until extraction.

#### 2.2.1. Preparation of Plant Extracts

The resulting powdered leaves were extracted using aqueous and ethanolic extraction methods following a modified method of Shahlehi et al. [21]. For aqueous extraction, 50 g of leaf powder was mixed with 500 mL of distilled water (1:10, *w*/*v*) and subjected to maceration in a 60 °C water bath (Stuart SBS40, Stone, Staffordshire, UK) for three days. The extraction was performed in two consecutive maceration cycles of three days each, under the same conditions using fresh solvent, resulting in a total extraction period of six days. The resulting extracts were combined and filtered, and the filtrate was centrifuged (Jouan BR4i, Saint-Herblain, France) at 3000 rpm for 20 min. The supernatant was stored at −80 °C overnight and subsequently freeze-dried (Labconco Freeze Dry System, Kansas City, MO, USA) for three days to yield a brown powdered extract. The freeze-dried extract was stored in an airtight container at 4 °C until further use.

For ethanolic extraction, ultrasonic extraction (UE) was employed. Briefly, 50 g of powdered leaves were mixed with 1000 mL of absolute ethanol (1:20, *w*/*v*) and subjected to sonication in a 300 W ultrasonic bath (Branson Ultrasonics, Danbury, CT, USA) at 25 °C for 30 min. After sonication, the extract was filtered through Whatman No. 1 filter paper (125 mm), and the filtrate was concentrated using a rotary evaporator (Yamato RE301, Tokyo, Japan) under reduced pressure (250 mbar) at 60 °C. The extraction procedure was repeated once using fresh solvent to maximize extraction efficiency, and the resulting filtrates were combined prior to evaporation. Following solvent removal, the concentrated extract was further dried in a drying cabinet at 60 °C for three days to obtain a dark-green solid extract. The dried ethanolic extract was stored in airtight containers at 4 °C until further use.

#### 2.2.2. Quantification of Extract Yield

The extraction yield of the leaf extracts for both solvents were calculated on a dry weight basis as the ratio of crude extract weight (in grams) to dry leaf powder before extraction weight (in grams), expressed as a percentage. The following equation calculated the extraction yield of *P. betle* leaves:

$$Yield\;(\%)=\frac{\mathrm{Mass}\mathrm{of}\mathrm{plant}\mathrm{crude}\mathrm{extract}\;(g)}{\mathrm{Mass}\mathrm{of}\mathrm{powder}\mathrm{plant}\mathrm{in}\mathrm{thimble}\;(g)}\times 100\%$$

### 2.3. Phytochemical Screening

Qualitative phytochemical screening of ethanolic and aqueous *P. betle* extracts was performed according to the protocol described by Shahlehi et al. [21]. Eleven phytochemical groups, including alkaloids, flavonoids, saponins, tannins, glycosides, steroids, and triterpenoids, were screened based on visual observation of color changes or precipitate formation following the addition of appropriate reagents. The results were recorded as present (+) or absent (−).

### 2.4. Total Phenolic and Total Flavonoid Contents Analysis

TPC was evaluated using Folin–Ciocalteu’s (FC) colorimetric assay according to the method described by [21], with minor modifications. Gallic acid was used as the reference standard for construction of the calibration curve (18.75, 37.5, 75, 150 and 300 μg/mL). Absorbance was measured at 765 nm using a microplate reader (BioTek Epoch, Winooski, VT, USA), and TPC was expressed as milligrams of gallic acid equivalents (mg GAE/g dry extract). Contrarily, TFC was determined using the aluminum chloride colorimetric method. Quercetin was used as the reference standard for preparation of the calibration curve (18.75, 37.5, 75, 150 and 300 μg/mL). Absorbance was measured at 415 nm using a microplate reader (BioTek Epoch, Winooski, VT, USA), and TFC was expressed as milligrams of quercetin equivalents (mg QE/g dry extract). For both assays, all analyses were performed in triplicates.

### 2.5. Gas Chromatography–Mass Spectrometry Analysis

Freshly prepared and stored ethanolic and aqueous extracts were characterized using gas chromatography–mass spectrometry (GC-MS) QP2010 (Shimadzu, Kyoto, Japan) to evaluate potential changes in phytochemical composition during storage. Fresh extracts were analyzed immediately after preparation, whereas stored extracts were analyzed following storage at 4 °C for 8 to 10 weeks. Ethanolic extracts were dissolved in methanol (GC-grade), while aqueous extracts were dissolved in distilled water, both to a final concentration of 1 mg/mL. The solutions were vortexed, filtered through a 0.45 μm syringe filter, and transferred into GC-MS autosampler vials. Samples (1 μL) were injected in split mode (10:1) with the injector temperature maintained at 250 °C. Chromatographic separation was performed on an SH-Rxi-5Sil MS capillary column (30 m × 0.25 mm i.d., 0.25 μm film thickness) using helium as the carrier gas at a constant column flow rate of 1.0 mL/min under a pressure of 53.5 kPa, with a linear velocity of 36.3 cm/s, total flow of 14.0 mL/min, and purge flow of 3.0 mL/min. The oven temperature was programmed from 50 °C (1 min hold) to 300 °C (rate 10 °C/min, 2 min hold at the final temperature). Mass spectra were acquired in electron ionization (EI) mode (70 eV) over a mass range of *m*/*z* 50–550, with the ion source and interface temperatures maintained at 200 °C and 280 °C, respectively, and a solvent cut time of 0 min. Compound identification was considered tentative and based solely on comparison of mass spectra with the NIST mass spectral library (SI ≥ 90). Retention indices, authentic standards and complementary analytical confirmation were not performed.

### 2.6. In Vitro Cell Culture

Human pancreatic ductal adenocarcinoma (PDAC) cell lines PANC-1, AsPC-1, Panc 02.03, Panc 04.03, Panc 08.13 and Panc 10.05, all of which harbor *KRAS* G12D mutations, were used in this study. The cells were maintained in RPMI-1640 (with L-glutamine) medium supplemented with FBS (heat inactivated, 10%) and kanamycin (25 mg/L). The cells were cultured in a humidified environment with 5% CO_2_ at 37 °C. The cells were subcultured upon reaching approximately 80% confluency.

#### WST-8 Cell Viability Assay

A WST-8 assay was performed to evaluate the effects of *P. betle* extracts on the viability of pancreatic cancer cell lines. PANC-1 (1 × 10^4^ cells/well), AsPC-1 (8 × 10^3^ cells/well), Panc 02.03 (8 × 10^3^ cells/well), Panc 04.03 (8 × 10^3^ cells/well), Panc 08.13 (2.5 × 10^4^ cells/well), and Panc 10.05 (3.5 × 10^4^ cells/well) cells were seeded in 96-well plates at their respective optimized seeding densities and incubated for 24 h to allow cell attachment. Seeding densities were determined through preliminary optimization experiments to achieve an absorbance value within the linear detection range of the WST-8 assay after 24 h of culture (OD > 0.5).

Based on the higher extraction yield, total phenolic content, total flavonoid content and more diverse phytochemical profile observed during preliminary characterization, the ethanolic *P. betle* extract was selected for subsequent biological evaluation. Cells were subsequently treated with serially diluted concentrations of ethanolic *P. betle* extracts (3–300 μg/mL). Vehicle (DMSO)-treated cells served as the normalization control for cell viability calculations, while Antimycin A (10, 30, and 100 μg/mL) was used as a positive technical control to confirm assay responsiveness. Following 24 h of treatment, WST-8 reagent was added to each well to a final concentration of 5% and incubated according to the manufacturer’s instructions. Absorbance was measured using a microplate reader, and cell viability was calculated relative to the DMSO-treated control:

$$\mathrm{Cell}\mathrm{viability}\;(\%)=\frac{OD(sample-blank)}{ODDMSO}\times 100\%$$

Concentration–response curves and half-maximal inhibitory concentration (IC_50_) values were generated from cell viability data. For ethanolic extracts, IC_50_ values were estimated by nonlinear regression using a four-parameter logistic (4PL) model.

### 2.7. Statistical Analyses

All experiments were performed in three independent biological replicates. Data are presented as mean ± standard error of mean (SEM). Prior to statistical analysis, normality was assessed using the Shapiro–Wilk test and homogeneity of variances was evaluated using the Brown–Forsythe test. Concentration–response curves and IC_50_ values for ethanolic extracts were generated by nonlinear regression using a 4PL model. The differences in cell viability between the DMSO-treated control and extract treatment across all cell lines were assessed using two-way analysis of variance (ANOVA) followed by Sidak’s multiple comparisons test. All statistical analyses were conducted using GraphPad Prism version 8.0.1 (GraphPad Software, Boston, MA, USA). A *p*-value of less than (<) 0.05 was considered statistically significant.

## 3. Results

### 3.1. Extraction Yield, Phytochemical Analysis, TFC, and TPC of P. betle Leaves Extract

The ethanolic extract of *P. betle* leaves displayed higher extraction yield than aqueous extract, at 17.26% relative to 11.96%, respectively. The resulting ethanolic extract was a viscous dark-green semi-solid, whereas the aqueous extract produced a light-brown powder-like consistency. Qualitative phytochemical screening of both the ethanolic and aqueous extracts revealed the presence of secondary metabolites, including flavonoids, steroids, terpenoids and carbohydrates, evident from resulting color changes (Table 1). Alkaloids and proteins were detected in the ethanolic extract but were not detected in the aqueous extract. Saponins, tannins, and glycosides were not detected in either extract.

Furthermore, both the TFC and TPC of the ethanolic extracts were higher than those of the aqueous extracts (Table 2). Despite the presence of flavonoids in qualitative testing, the TFC of the aqueous extracts were found to be lower than that of the ethanolic extracts, at 84.20 ± 0.002 mg QE/g and 121.28 ± 0.009 mg QE/g respectively. The TFC values of the ethanolic and aqueous extracts were calculated using a quercetin calibration curve obtained at y = 0.0026x − 0.0156 (R^2^ = 0.9977) and y = 0.0009x − 0.0164 (R^2^ = 0.9809), respectively.

Similarly, the ethanolic extracts displayed remarkable TPC value, at 395.94 ± 0.010 mg GAE/g in comparison to the aqueous extracts at 186.47 ± 0.009 mg GAE/g, though this has shown the presence of phenols in the aqueous extracts. The TPC value was calculated using the gallic acid calibration curve acquired at y = 0.0021x − 0.001 (R^2^ = 0.999) for the ethanol extracts and y = 0.0027x + 0.0164 (R^2^ = 0.9956) for the aqueous extracts. Nevertheless, both the ethanolic and aqueous extracts exhibited flavonoid and phenolic contents, relevant for anticancer activity.

### 3.2. GC-MS Analysis of P. betle Leaf Extracts

**Figure 1:**
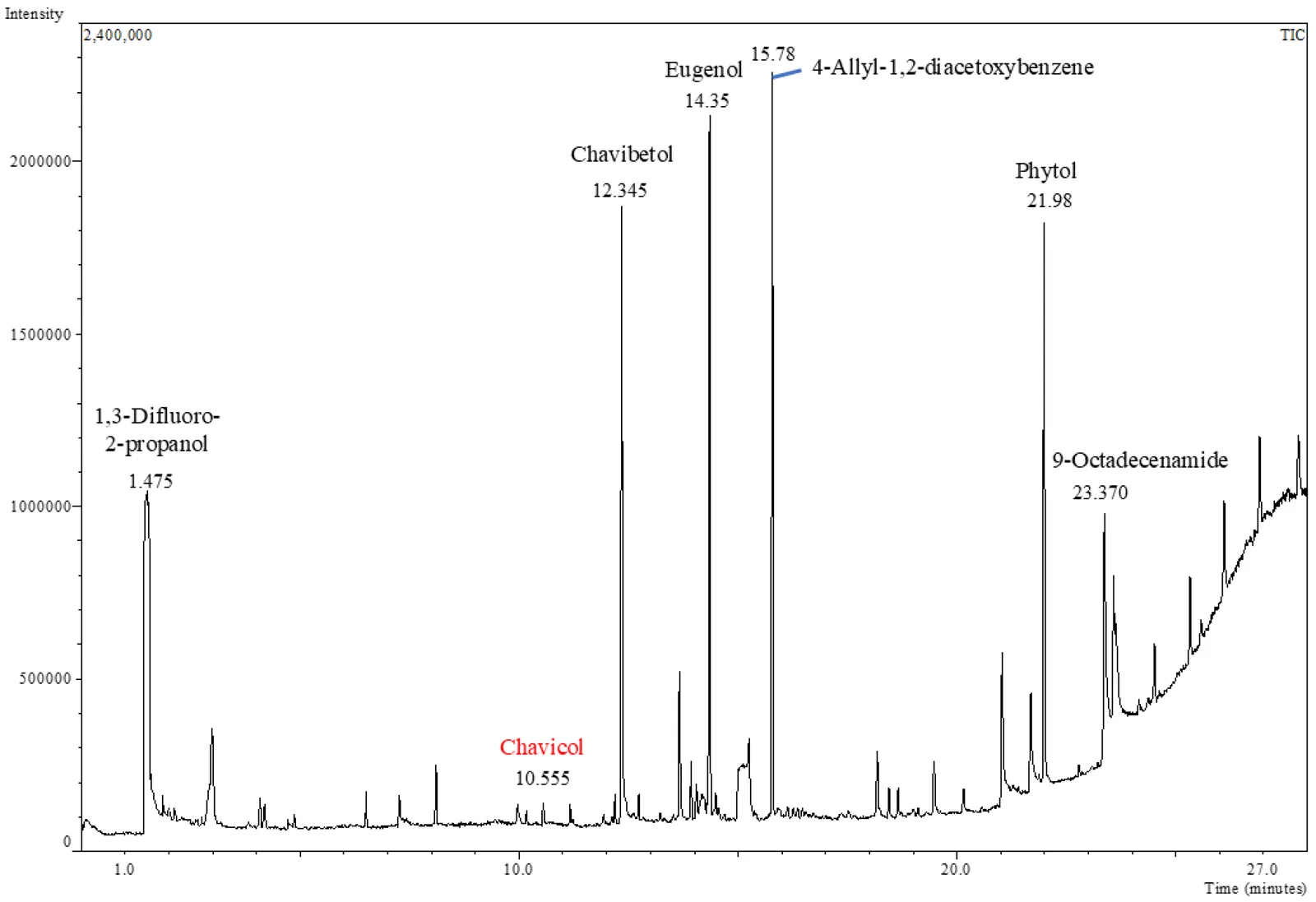
GC–MS chromatogram of freshly prepared ethanolic *Piper betle* leaf extracts. The major peaks correspond to the tentatively assigned compounds identified by comparison of mass spectra with the NIST mass spectral library (similarity index ≥ 90).

**Figure 2:**
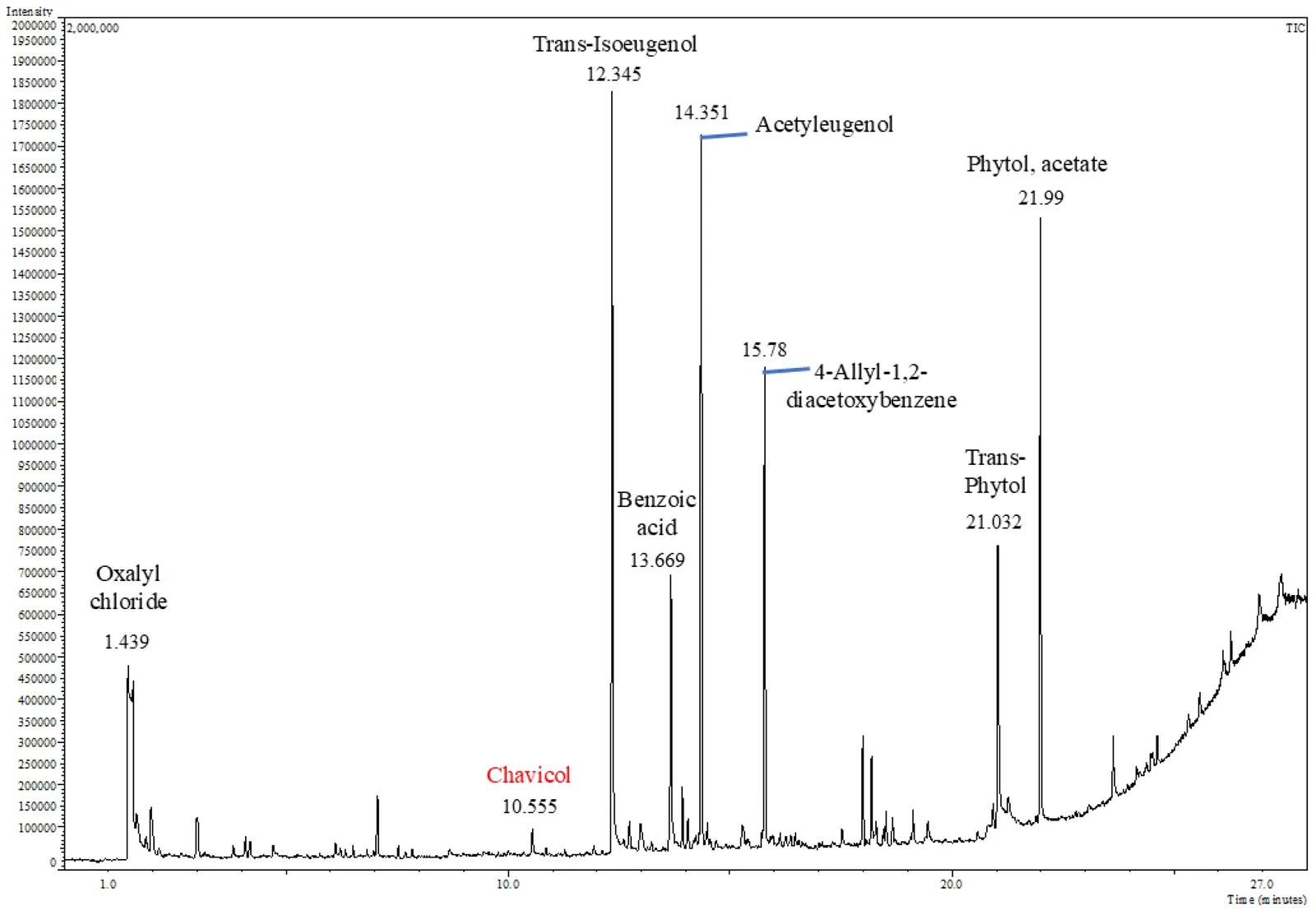
GC–MS chromatogram of stored ethanolic *Piper betle* leaf extracts. The major peaks correspond to the tentatively assigned compounds identified by comparison of mass spectra with the NIST mass spectral library (similarity index ≥ 90).

Based on tentative GC-MS findings, the ethanolic extracts exhibited a more diverse chromatographic profile than the aqueous extracts. Major tentatively assigned compounds include chavicol, chavibetol, eugenol, trans-isoeugenol, acetyleugenol, phytol, and phytol acetate. Chavicol was tentatively detected in both freshly prepared and stored ethanolic extracts, while chavibetol and eugenol were observed mainly in freshly prepared extracts (Table 3; Figure 1 and Figure 2). In contrast, acetyleugenol and trans-isoeugenol (derivatives of eugenol) were detected only in stored extracts.

**Figure 3:**
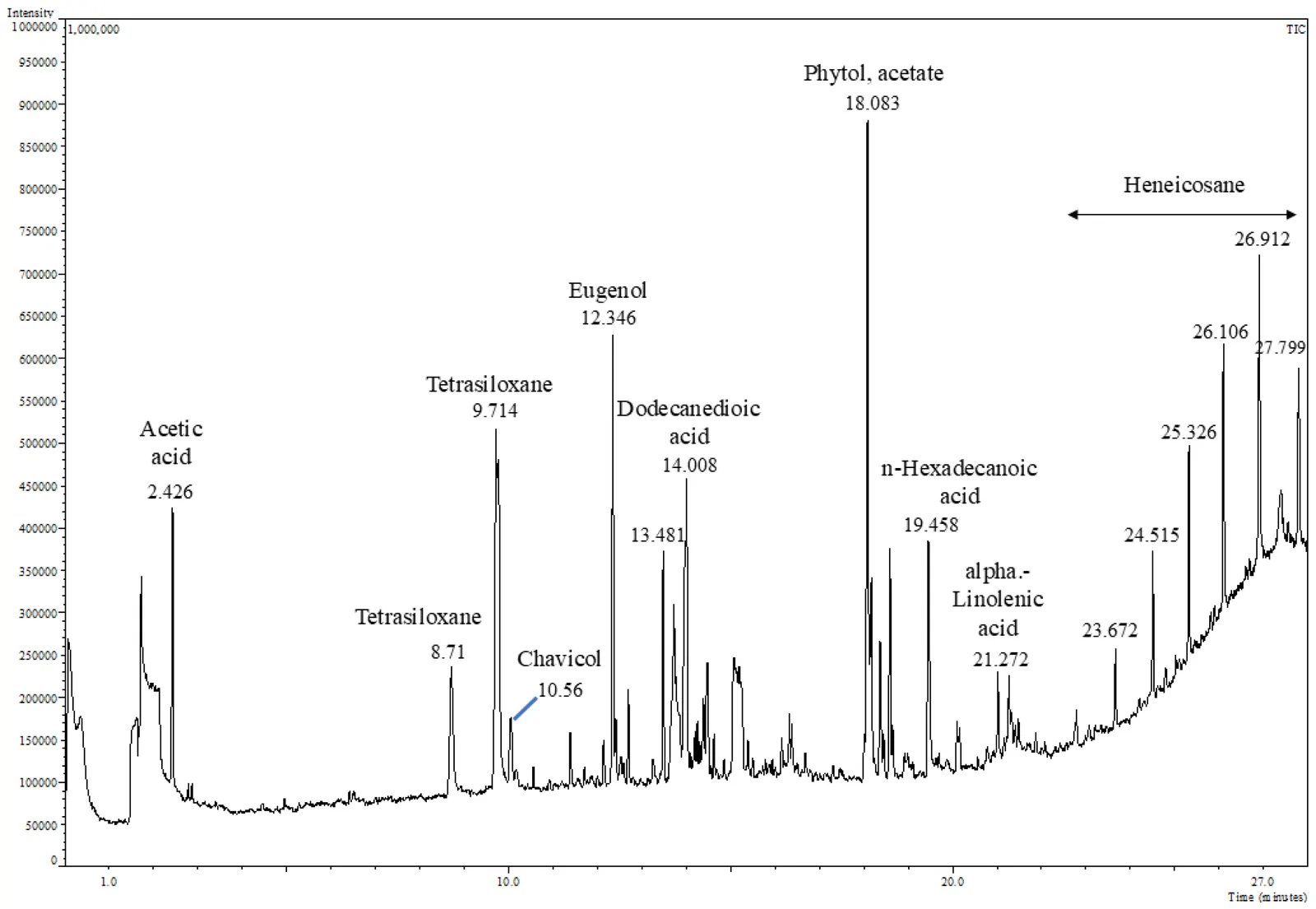
A GC–MS chromatogram of freshly prepared aqueous *Piper betle* leaf extracts. The major peaks correspond to the tentatively assigned compounds identified by comparison of mass spectra with the NIST mass spectral library (similarity index ≥ 90).

**Figure 4:**
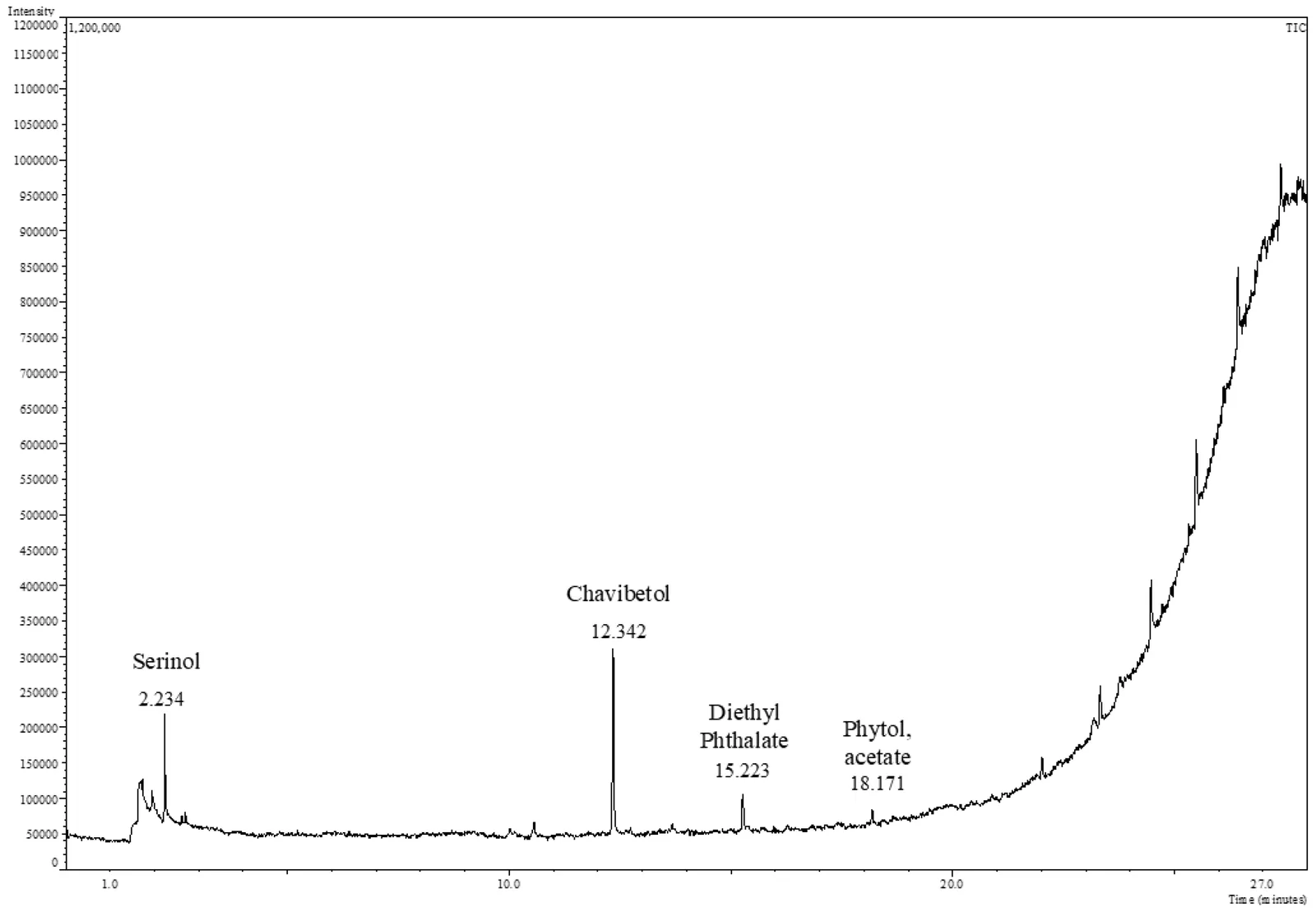
A GC–MS chromatogram of stored aqueous *Piper betle* leaf extracts. The major peaks correspond to the tentatively assigned compounds identified by comparison of mass spectra with the NIST mass spectral library (similarity index ≥ 90).

In contrast, the aqueous extracts exhibited a comparatively less complex phytochemical profile. Tentatively assigned compounds included serinol, chavicol, chavibetol, phytol acetate and heneicosane. Chavibetol was detected in freshly prepared aqueous extracts, whereas chavicol and heneicosane were detected in stored aqueous extracts (Table 4; Figure 3 and Figure 4).

Overall, the ethanolic extract yielded a greater diversity of tentatively identified phenolic and phenylpropanoid compounds compared with the aqueous extract. Chavicol, chavibetol, and eugenol were detected in both the ethanolic and aqueous extracts, whereas compounds such as acetyleugenol and trans-isoeugenol were observed predominantly in the ethanolic extracts under the analytical conditions employed in this study.

### 3.3. Concentration-Dependent Reduction in Cell Viability Following Treatment with P. betle Ethanolic Leaf Extracts

**Figure 5:**
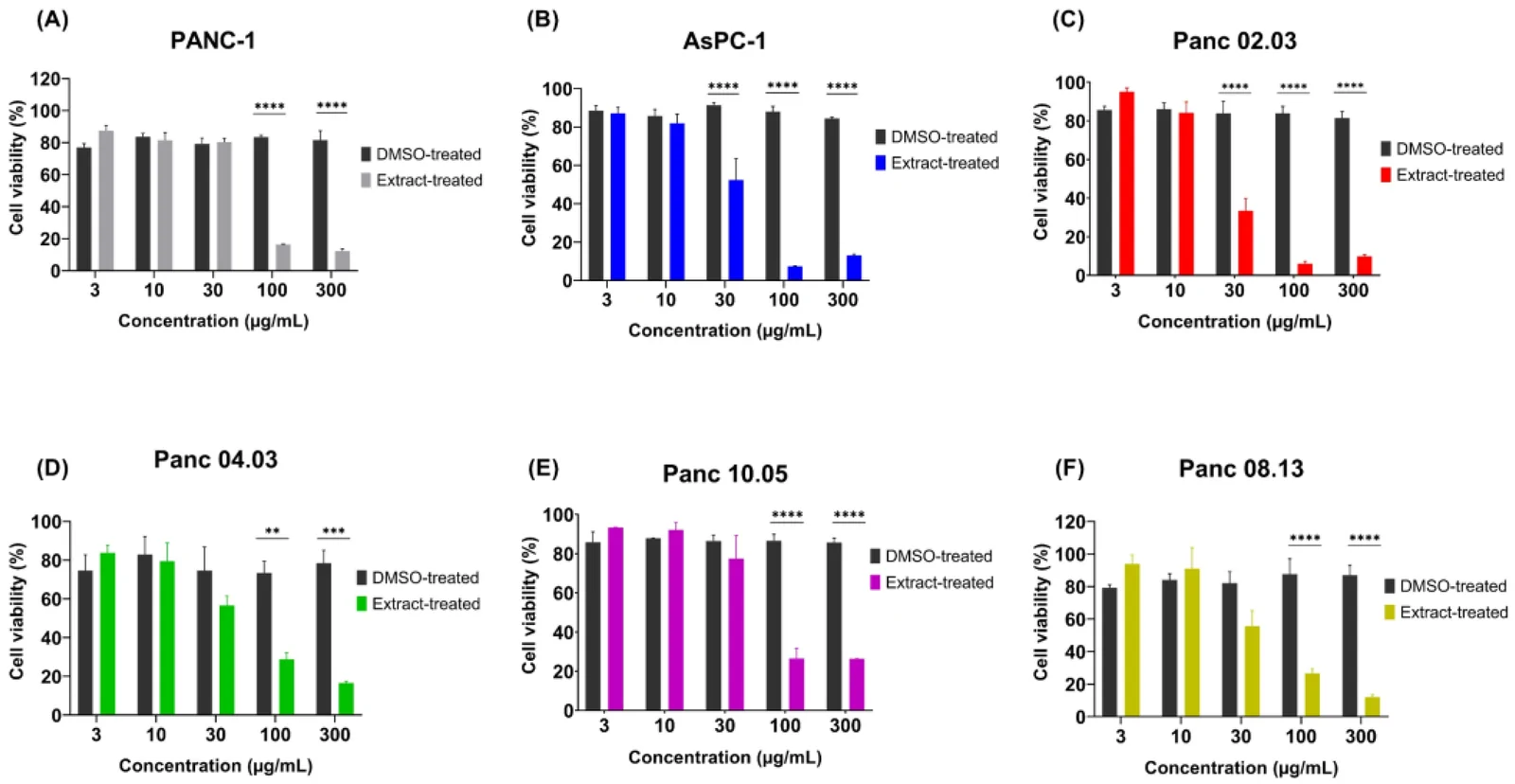
The concentration-dependent effects of ethanolic *P. betle* leaf extract on the viability of human *KRAS* G12D-mutant pancreatic cancer cell lines. (**A**) PANC-1, (**B**) AsPC-1, (**C**) Panc 02.03, (**D**) Panc 04.03, (**E**) Panc 10.05, and (**F**) Panc 08.13 were treated with increasing concentrations of ethanolic extract (3, 10, 30, 100, and 300 µg/mL) for 24 h. Cell viability was assessed using the WST-8 assay and expressed as percentage relative to the DMSO-treated control. The data are presented as mean ± SEM from at least three independent experiments. Statistical significance was determined using two-way ANOVA followed by Sidak’s multiple comparisons analysis. The asterisks indicate significant differences between the DMSO-treated and extract-treated cells at the corresponding concentration (** *p* < 0.01, *** *p* < 0.001, and **** *p* < 0.0001).

Considering its higher extraction yield, greater total phenolic and flavonoid contents, and richer phytochemical profile, the ethanolic extract was selected for subsequent biological evaluation. Treatment with ethanolic leaf extracts of *P. betle* reduced the viability of *KRAS* G12D-mutant pancreatic cancer cell lines in a concentration-dependent manner. Cell viability progressively decreased with increasing extract concentration. Significant reductions in cell viability were first observed at 30 μg/mL in the AsPC-1 and Panc 02.03 cells (*p* < 0.0001), whereas PANC-1, Panc 04.03, Panc 10.05, and Panc 08.13 exhibited significant reductions starting at 100 μg/mL (*p* < 0.01 and *p* < 0.0001) (Figure 5).

#### Response of KRAS G12D-Mutant Pancreatic Cancer Cell Lines to Ethanolic *Piper betle* Extract

**Figure 6:**
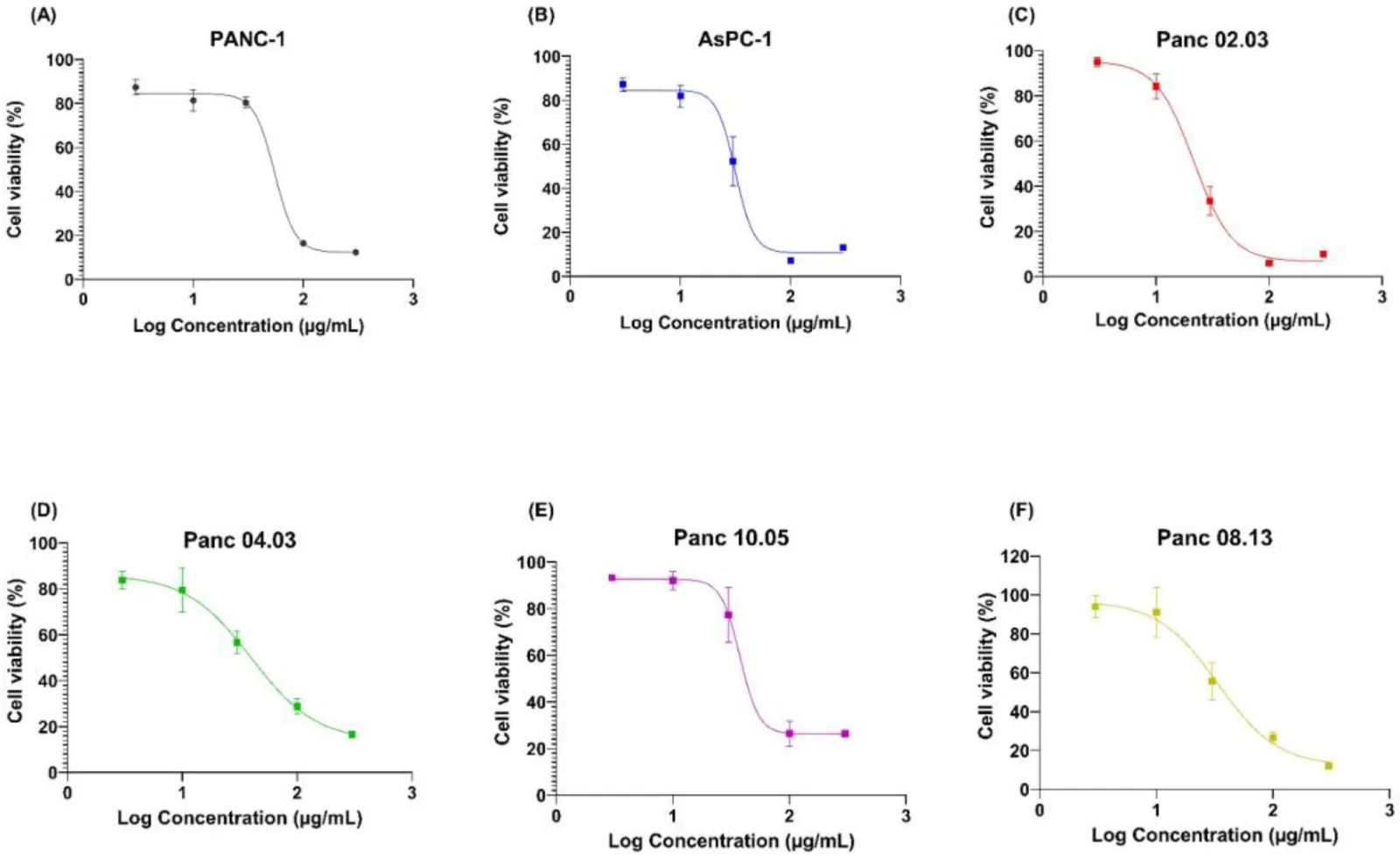
The concentration–response curves of pancreatic cancer cell lines following treatment with ethanolic *P. betle* extract. (**A**) PANC-1, (**B**) AsPC-1, (**C**) Panc 02.03, (**D**) Panc 04.03, (**E**) Panc 10.05, and (**F**) Panc 08.13. The cells were treated with ethanolic *P. betle* extract (3–300 μg/mL) for 24 h and cell viability was determined using the WST-8 assay. Cell viability is expressed as a percentage relative to the DMSO-treated control. The concentration–response curves were generated by nonlinear regression using a four-parameter logistic (4PL) model. The data are presented as mean ± SEM of three independent experiments (*n* = 3).

Concentration–response curves generated using nonlinear regression demonstrated a progressive reduction in cell viability following treatment with the ethanolic *P. betle* leaf extract across the cell lines tested (Figure 6). Estimated IC_50_ values ranged from 21.54 to 54.91 µg/mL across the six *KRAS* G12D-mutant pancreatic cancer cell lines. Among the cell lines evaluated, Panc 02.03 cells exhibited the lowest IC_50_ value (21.54 µg/mL), whereas PANC-1 cells displayed the highest estimated IC_50_ value (54.91 µg/mL), indicating comparatively lower sensitivity to the ethanolic extract (Table 5). Overall, the ethanolic extract demonstrated inhibitory activity across all *KRAS* G12D-mutant pancreatic cancer cell lines within a relatively narrow IC_50_ range.

## 4. Discussion

This study investigated the phytochemical composition and cytotoxic activity of ethanolic and aqueous extracts of *P. betle* leaves against human *KRAS* G12D-mutant pancreatic cancer cell lines, including PANC-1, AsPC-1, Panc 02.03, Panc 04.03, Panc 08.13 and Panc 10.05. Overall, the ethanolic extract demonstrated higher extraction yield, greater total phenolic and flavonoid contents and a more diverse phytochemical profile than the aqueous extract. Based on these preliminary phytochemical characteristics, the ethanolic extract was selected for subsequent biological evaluation, where it produced a concentration-dependent reduction in cell viability across all pancreatic cancer cell lines examined, supporting the potential of *P. betle* as a source of bioactive compounds with antiproliferative activity against PDAC cells.

The higher extraction yield and increased TPC and TFC values obtained from the ethanolic extract are consistent with previous reports showing that ethanol, which is less polar than water, allows the extraction of a distinct profile of bioactive compounds such as phenolic and phenylpropanoid compounds from *P. betle* [22,23,24]. Phenolics and flavonoids are known to possess antioxidants, anti-inflammatory and anticancer properties through the modulation of oxidative stress, inhibition of tumor cell proliferation and induction of apoptosis [25,26,27]. Although aqueous extracts also contained detectable phenolics and flavonoids, their overall phytochemical content was considerably lower. Because the ethanolic and aqueous extracts were prepared using different extraction techniques, extraction durations and temperatures, these differences should not be attributed solely to solvent polarity. Rather, the preliminary phytochemical analyses were used to guide the selection of the ethanolic extract for subsequent biological evaluation.

GC-MS analysis further supported these findings by revealing a greater diversity of tentatively identified phenolic and phenylpropanoid compounds in the ethanolic extract than in the aqueous extract. The tentatively assigned constituents included chavicol, chavibetol, eugenol, trans-isoeugenol, acetyleugenol, phytol and phytol acetate. Chavicol was detected in both freshly prepared and stored ethanolic extracts, whereas trans-isoeugenol and acetyleugenol were detected predominantly after storage, suggesting minor qualitative changes in extract composition during storage. However, because compound assignments were based solely on NIST mass spectral library matching and no quantitative analysis, retention index determination or authentic standard confirmation was performed, these observations should be interpreted cautiously. The apparent compositional differences may reflect storage-related chemical transformations, analytical variability, or differences in sample preparation, and dedicated stability studies are required to confirm these findings. Nevertheless, these tentatively identified compounds have previously been reported as characteristic constituents of *P. betle* and are associated with antioxidant, antimicrobial and antiproliferative activities [18,20,28,29].

The ethanolic *P. betle* extract produced a concentration-dependent reduction in cell viability across all pancreatic cancer cell lines investigated, with estimated IC_50_ values ranging from 21.54 to 54.91 µg/mL. These findings indicate that the extract possesses inhibitory activity against multiple PDAC cell lines despite differences in their molecular backgrounds. Comparable antiproliferative activity has been reported previously for *P. betle*-derived compounds. Guha Majumdar and Subramanian [30] demonstrated that hydroxychavicol, a major phenolic constituent of *P. betle*, inhibited colony formation and reduced the viability of PANC-1 and MIA PaCa-2 pancreatic cancer cells, with IC_50_ values of approximately 22 and 30 µM, respectively. Similarly, Lee et al. [31] reported that crude *P. betle* stem extract reduced the viability of Cal-27 and Ca9-22 oral squamous carcinoma cells in a concentration-dependent manner, with IC_50_ values of 130.6 and 133.4 µg/mL, respectively. Although there are differences in plant source, extraction method, extract composition, assay conditions and cancer models, the relatively low IC_50_ values obtained in the present study support the potential biological activity of ethanolic *P. betle* extracts against pancreatic cancer cells.

The concentration-dependent reduction in cell viability observed following ethanolic *P. betle* extract treatment suggests that the extract may interfere with cellular proliferation and survival pathways. Previous studies have demonstrated that *P. betle* extracts and their major phenolic constituents, including eugenol, chavicol and chavibetol, are capable of inducing apoptosis through activation of caspase-dependent pathways and generation of reactive oxygen species [14,15,19,30]. In addition, these compounds have been reported to suppress cell-cycle progression and inhibit signaling pathways associated with tumor growth [30,31]. Although the present study evaluated only cell viability using the WST-8 assay, similar processes may contribute to the inhibitory effects observed in pancreatic cancer cells. Additional mechanistic studies incorporating apoptosis assays, reactive oxygen species measurements, cell-cycle analysis and investigations of *KRAS*-associated signaling pathways will be required to determine the mechanisms underlying the observed biological activity.

The main objective of this study was to investigate the response of *KRA*S G12D-mutant pancreatic cancer cells to *P. betle* ethanolic extracts. Although the observed differences were not statistically significant, *KRAS* G12D-mutant cell lines appeared to exhibit great susceptibility to the ethanolic extracts. This trend may suggest that certain *KRAS* G12D-mutant pancreatic cancer cell lines are more responsive to *P. betle* extracts; however, the limited statistical significance observed indicates that this finding should be interpreted with caution. Previous studies have shown that *KRAS*-mutant cancers undergo extensive metabolic reprogramming and exhibit increased dependence on signaling pathways such as MAPK/ERK and PI3K/AKT for survival and proliferation [7,12]. One possible explanation is that *KRAS-*mutant cells exhibit altered redox regulation and metabolic dependencies, which may influence their response to phytochemical compounds. Therefore, further mechanistic studies including the incorporation of appropriate *KRAS* wild-type pancreatic cancer cells and non-malignant pancreatic ductal epithelial cells will be required to determine whether mutation-specific or cancer-selective responses exist.

From a translational perspective, Antimycin A was included only as a positive technical control to confirm assay responsiveness and was not intended as a clinically relevant therapeutic comparator. Future investigations should therefore compare *P. betle* extracts or isolated bioactive constituents with established PDAC therapies such as gemcitabine, FOLFIRINOX-associated agents, or emerging *KRAS* G12D-targeted inhibitors. In particular, the highly selective non-covalent *KRAS* G12D inhibitor MRTX1133 has demonstrated more than 1000-fold selectivity for *KRAS* G12D over wild-type *KRAS* and has produced substantial tumor regression in preclinical PDAC models [32]. Such comparisons would provide a more clinically relevant assessment of the therapeutic potential of *P. betle*-derived compounds and help position them as potential adjunctive or combination therapeutic candidates.

Several limitations should be considered when interpreting the findings of this study. The GC-MS analysis performed was qualitative and compound assignments were tentative because identification relied solely on NIST library matching without retention index determination or confirmation using authentic standards. Future studies should therefore incorporate retention index determination, authentic standards and complementary analytical approaches, such as LC–MS, to improve confidence in compound identification. In addition, only a single extraction batch was evaluated; therefore, batch-to-batch variability in phytochemical composition and biological activity was not assessed. Standardized extraction procedures and evaluation of multiple extraction batches will be needed to improve reproducibility. Furthermore, the WST-8 assay evaluated only cell viability and did not investigate the mechanisms between growth inhibition, reduced metabolism, and cell death. The effects of the extracts on non-malignant pancreatic cells or *KRAS* wild-type pancreatic cancer cells were also not evaluated, preventing assessment of mutation-specific responses and cancer selectivity. Finally, comparisons with clinically relevant pancreatic cancer therapeutics, such as gemcitabine, FOLFIRINOX or *KRAS* G12D-targeted inhibitors including MRTX1133, together with in vivo efficacy and safety studies, will be essential to establish the translational potential of *P. betle*-derived compounds [9,33]. Future studies should therefore focus on isolating and characterizing the active compounds responsible for the observed effects and evaluating their influence on apoptosis, cell-cycle regulation and *KRAS*-signaling pathways, including MAPK/ERK and PI3K/AKT.

## 5. Conclusions

In conclusion, ethanolic *P. betle* leaf extract demonstrated a more diverse phytochemical composition, higher phenolic and flavonoid contents, and concentration-dependent inhibitory activity against human pancreatic cancer cell lines, including *KRAS* G12D-mutant models. GC–MS analysis tentatively identified several phenolic and phenylpropanoid compounds, including chavicol, chavibetol, eugenol, trans-isoeugenol, acetyleugenol, phytol and phytol acetate, which may collectively contribute to the observed biological activity. Although inhibitory effects were observed across all *KRAS* G12D-mutant pancreatic cancer cell lines examined, these do not imply evidence of mutation-specific sensitivity. Overall, these findings support the potential of *P. betle* as a promising source of bioactive compounds for pancreatic cancer research. Future studies should focus on phytochemical standardization, mechanistic investigations, evaluation in *KRAS* wild-type and non-malignant pancreatic cell models, and preclinical in vivo validation to further assess its therapeutic potential.

## Figures and Tables

**Table 1:** Preliminary phytochemical screening of ethanol and aqueous extracts of *P. betle* leaves.

Phytochemical Group	Ethanol	Aqueous
Alkaloids	++	−
Flavonoids	++	++
Steroids	+++	+++
Terpenoids	++++	+++
Protein	+++	−
Carbohydrates	+++	+++
Tannins	−	−
Saponins	−	−
Glycosides	−	−

Note: The following represents the relative abundance of phytochemicals in plant extracts: ++++ denotes a very high presence, +++ a high presence, ++ a moderate presence, and − indicates absence.

**Table 2:** TPC and TFC of *P. betle* leaf extracts.

Extract	TFC (mg QE/g)	TPC (mg GAE/g)
Ethanol	121.28 ± 0.009	395.94 ± 0.010
Aqueous	84.20 ± 0.002	186.47 ± 0.009

Note: Values are expressed as mean of mg of GAE or QE per gram of extract ± SEM; *n* = 3. GAE: gallic acid equivalent; QE: quercetin equivalent; ± SEM: standard error of the mean.

**Table 3:** Compounds tentatively assigned in ethanolic *Piper betle* leaf extracts by GC-MS.

Extract	RT (min)	Compound	Molecular Formula	Chemical Structure	MW	SI
Fresh	12.345	Chavibetol	C_10_H_12_O_2_	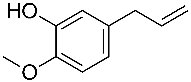	164	96
	12.35	Eugenol *	C_10_H_12_O_2_	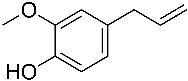	164	95
Fresh/Stored	10.55	Chavicol	C_9_H_10_O	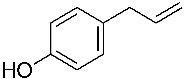	134	91–92
	21.99	Phytol acetate	C_22_H_42_O_2_	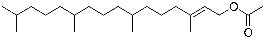	338	93
Stored	12.35	Trans-isoeugenol	C_10_H_12_O_2_	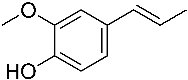	164	91–95
	14.35	Acetyleugenol	C_12_H_14_O_3_	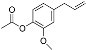	206	95

Note: * Chavibetol and eugenol were obtained as alternative library matches for the same chromatographic peak and were not confirmed by authentic standards. RT, retention time; MW, molecular weight (g/mol); SI, similarity index obtained from comparison with the NIST mass spectral library. Only compounds with SI ≥ 90 are shown. The compounds were identified in fresh and/or stored ethanolic *P. betle* extracts. Fresh/Stored indicates compounds tentatively detected in both freshly prepared and stored extracts.

**Table 4:** Compounds tentatively identified in aqueous *Piper betle* leaf extracts by GC-MS.

Extract	RT (min)	Compound	Molecular Formula	Chemical Structure	MW	SI
Fresh	2.24	Serinol	C_3_H_9_NO_2_	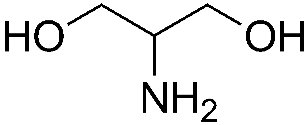	91	94
	12.346	Eugenol	C_10_H_12_O_2_	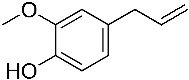	164	
Fresh/Stored	18.10	Phytol acetate	C_22_H_42_O_2_	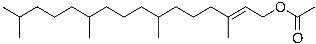	338	93
Stored	10.56	Chavicol	C_9_H_10_O	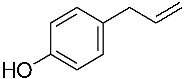	134	90
	12.35	Chavibetol	C_10_H_12_O_2_	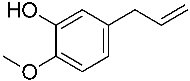	164	95–96
	25.33	Heneicosane	C_21_H_44_	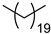	296	94

Note: RT, retention time; MW, molecular weight (g/mol); SI, similarity index obtained from comparison with the NIST mass spectral library. Only compounds with SI ≥ 90 are shown. The compounds were tentatively identified in fresh and/or stored aqueous *P. betle* extracts.

**Table 5:** Estimated IC_50_ values of ethanolic *P. betle* extract against *KRAS* G12D-mutant pancreatic cancer cells.

Cell Line	Estimated IC_50_ (µg/mL)	R^2^
PANC-1	54.91	0.9812
AsPC-1	31.49	0.9344
Panc 02.03	21.54	0.9747
Panc 04.03	39.84	0.9333
Panc 10.05	37.61	0.9119
Panc 08.13	33.47	0.9378

Note: IC_50_ values were estimated by nonlinear regression using a 4PL model. R^2^ values indicate the goodness-of-fit of each fitted curve.

## Data Availability

The original contributions presented in this study are included in the article. Further inquiries can be directed to the corresponding author.

## Abbreviations

The following abbreviations are used in this manuscript:

AKT	Protein kinase B
ANOVA	Analysis of variance
ATCC	American Type Culture Collection
CO_2_	Carbon dioxide
DMSO	Dimethyl sulfoxide
EI	Electron ionization
EtOH	Ethanol/Ethanolic extract
FBS	Fetal bovine serum
FC	Folin–Ciocalteu
FOLFIRINOX	Combination chemotherapy regimen (folinic acid, fluorouracil, irinotecan, oxaliplatin)
GAE	Gallic acid equivalent
GC-MS	Gas chromatography–mass spectrometry
H_2_O	Aqueous extract
IC_50_	Half-maximal inhibitory concentration
KRAS	Kirsten rat sarcoma viral oncogene homolog
MAPK	Mitogen-activated protein kinase
MW	Molecular weight
NIST	National Institute of Standards and Technology
PDAC	Pancreatic ductal adenocarcinoma
PI3K	Phosphoinositide 3-kinase
QE	Quercetin equivalent
RIKEN BRC	RIKEN BioResource Research Center
RPMI	Roswell Park Memorial Institute
RT	Retention time
R^2^	Goodness-of-fit
SEM	Standard error of the mean
SI	Similarity index
TFC	Total flavonoid content
TPC	Total phenolic content
UE	Ultrasonic extraction
WST-8	Aqueous-soluble tetrazolium salt-8
